# Associations of mortality with own height using son's height as an instrumental variable

**DOI:** 10.1016/j.ehb.2012.04.003

**Published:** 2013-07

**Authors:** David Carslake, Abigail Fraser, George Davey Smith, Margaret May, Tom Palmer, Jonathan Sterne, Karri Silventoinen, Per Tynelius, Debbie A. Lawlor, Finn Rasmussen

**Affiliations:** a MRC Centre for Causal Analyses in Translational Epidemiology (CAiTE), School of Social and Community Medicine, University of Bristol, United Kingdom; b School of Social and Community Medicine, University of Bristol, United Kingdom; c Population Research Unit, Department of Social Research, University of Helsinki, Helsinki, Finland; d Child and Adolescent Public Health Epidemiology, Department of Public Health Sciences, Karolinska Institute, Stockholm, Sweden

**Keywords:** IV, instrumental variable, HR, hazard ratio, SD, standard deviation, CI, confidence interval, CVD, cardiovascular disease, BMI, body mass index, SEP, socioeconomic position, CHD, coronary heart disease, Body height, Mortality, Confounding factor, Causality, Cohort studies

## Abstract

Height is associated with mortality from many diseases, but it remains unclear whether the association is causal or due to confounding by social factors, genetic pleiotropy,^1^ or existing ill-health. The authors investigated whether the association of height with mortality is causal by using a son's height as an instrumental variable (IV) for parents’ height among the parents of a cohort of 1,036,963 Swedish men born between 1951 and 1980 who had their height measured at military conscription, aged around 18, between 1969 and 2001. In a two-sample IV analysis adjusting for son's age at examination and secular trends in height, as well as parental age, and socioeconomic position, the hazard ratio (HR) for all-cause paternal mortality per standard deviation (SD, 6.49 cm) of height was 0.96 (95% confidence interval (CI): 0.95, 0.96). The results of IV analyses of mortality from all causes, cardiovascular disease (CVD), respiratory disease, cancer, external causes and suicide were comparable to those obtained using son's height as a simple proxy for own height and to conventional analyses of own height in the present data and elsewhere, suggesting that such conventional analyses are not substantially confounded by existing ill-health.

## Highlights

► Associations of exposures with mortality may be confounded by existing ill health. ► We used a son's height as an instrument for parents’ height to avoid confounding. ► Parents of taller sons had lower cardiovascular and respiratory disease mortality. ► Parents of taller sons had higher mortality from cancer. ► Previous studies of height are not substantially confounded by existing ill health.

## 1 Introduction

Predominantly inverse associations of height with all-cause mortality have been found in a large number of studies in developed countries (Davey Smith et al., 2000; Engeland et al., 2003; Jousilahti et al., 2000; Koch, 2011; Song et al., 2003; Song and Sung, 2008). This appears to be driven by inverse associations of height with cardiovascular disease (CVD) and respiratory disease mortality (Cook et al., 1994; Gunnell et al., 2003; Lawlor et al., 2004; Lee et al., 2009; Paajanen et al., 2010), and partly counteracted by positive associations with many forms of non-smoking related cancers (Batty et al., 2010; Green et al., 2011; Gunnell et al., 2001). The mechanisms underlying these associations, however, are unclear. They might be explained by residual confounding, for example by socioeconomic position (Batty et al., 2006; Davey Smith et al., 2000; Gunnell, 2002; Leon et al., 1995), by genetic variants with pleiotropic effects (Silventoinen et al., 2003), or confounding due to existing but undiagnosed illness (sometimes also referred to as reverse causality). Beyond confounding, a range of possible biological mechanisms have been proposed. For example, the greater lung capacity of taller people might be protective against respiratory disease (Batty et al., 2006; Davey Smith et al., 2000), while the increased risk of most cancers in taller people might be due to the increased number of cells available to become cancerous (Albanes and Winick, 1988). It is important to establish whether an association is causal before exploring the plausibility of different biological mechanisms which could provide insights into the prevention of disease.

Various approaches have been taken to quantify, or to adjust for, the influence of confounding on these associations. Studies of monozygotic and dizygotic twins (Lundqvist et al., 2007; Silventoinen et al., 2003, 2006) can begin to establish whether genetic or shared environmental characteristics explain the associations. The study of socially homogeneous groups (McCarron et al., 2002; Murray, 1997; Okasha et al., 2000) has reduced the influence of social confounding, at the cost of a loss of generality. To avoid confounding due to ill-health and the resultant shrinkage, several studies have used a subject's maximum height, or excluded deaths occurring in the first few years after measurement (Batty et al., 2010; Leon et al., 1995; McCarron et al., 2002). These studies suggest that all of the listed sources of confounding may apply for some causes of mortality in some circumstances, but adjustment for confounders has always been imperfect, and many studies have been restricted to particular causes of mortality, in limited study samples.

Here, we take another approach to adjustment for confounding, using offspring height as an instrumental variable (IV) for assessing the causal effect of own height on all-cause and cause-specific mortality. The adult height of a subject's offspring is strongly correlated with the subject's own height, but is unlikely to be substantially influenced by the subject's existing, but undiagnosed illness. The latter assertion is untestable, but we argue that it is plausible since (i) much of the variation in height is genetically determined and (ii) most parental illness and death occurs after children reach adult height. Thus, we can use offspring height as an IV for own height, under the assumption that this will better control for confounding by existing illness, though we acknowledge that this approach will not fully control for confounding by socioeconomic, lifestyle or genetic characteristics which are shared by parents and offspring. We have previously used this approach to examine the causal effect of BMI on all-cause and cause-specific mortality (Davey Smith et al., 2009).

## 2 Study population and data linkage

**Fig. 1:**
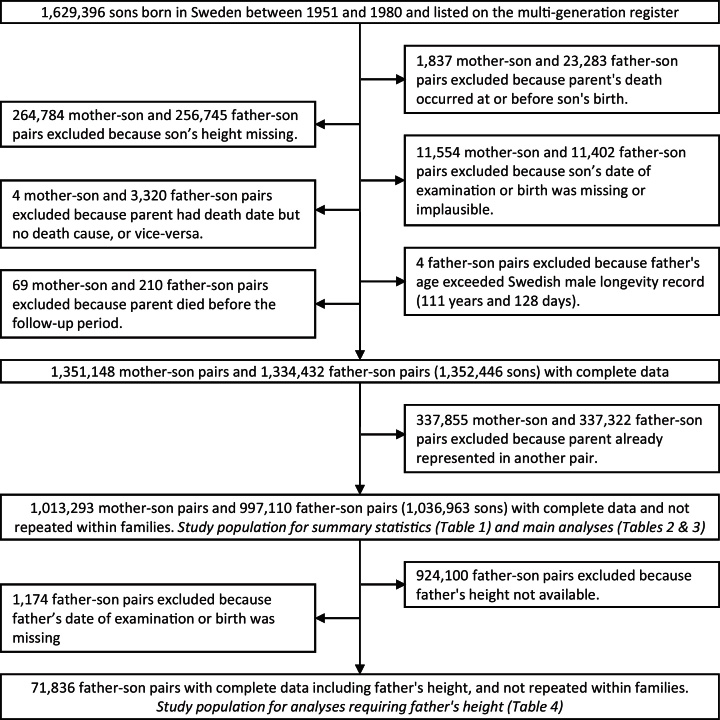
Flow of participants through the study; parents of Swedish men born between 1951 and 1980 and subjected to conscription examination.

The unique national identity numbers and dates of birth of all 1,629,396 boys born in Sweden between 1951 and 1980, and of their biological parents, were extracted from the Swedish Multi-Generation Register (Fig. 1). The sons’ identity numbers were used to obtain their height, where available, from records of conscription examinations between September 1969 and December 2001. Weight and blood pressure were also available from most examinations, and smoking habits were available from 29,541 examinations, mostly in 1969 or 1970. Conscription examinations were compulsory for young Swedish men during the study period, except those with severe handicap or chronic disease, and took place at a mean age of 18.3 years (range 16–25, with 91% aged 17 or 18). The 16% with missing data were primarily due to accidental loss following changes in data management at the conscription authority. To avoid pseudo-replication within families, only one son from each parent was retained in the data set. Sons to be retained were randomly chosen, except that the same son was retained for both of his parents whenever possible.

Parents’ identity numbers were matched to the Swedish Cause of Death Register, providing the underlying cause of death for all deaths between 1961 and 2004. Emigration records were also available, allowing the assumption that parents not dead or emigrated by 31 December 2004 were still alive at this time. The conscription examination records included data for 71,836 father–son pairs, with fathers having undergone examinations between 1969 and 1991. The Swedish Population and Housing Census provided data on parents’ educational level and occupational socioeconomic index (SEI) in 1970 and 1990. We took the higher of the 1970 and 1990 values for educational level and classified it into five levels: <9 years; 9–10 years; full secondary; higher; and missing (2% of mothers and 5% of fathers). We also classified parents according to five mutually exclusive categories of occupational SEI: high/intermediate non-manual; low non-manual; skilled manual; unskilled manual; and other/missing. We used the 1970 value for parents born before 1935 and the 1990 value for parents born later. 27% of mothers fell into the other/missing category, comprising 2% missing data and 25% others (including housewives, farmers (<1%) and part-time workers). 20% of fathers were categorised as other/missing, comprising 2% missing, 4% farmers and 14% others. Cause-specific deaths (see Table A1 in Appendix) were defined using the international classification of diseases codes (ICD versions 7–10) from the cause of death register, with some causes being subsets of others (for example, site specific cancers were subsets of cancer as a whole), in a similar manner to Davey Smith et al. (2009).

Our final main analyses were conducted on 997,110 father–son pairs and 1,013,293 mother–son pairs, with additional analyses that used father's height as well as son's height conducted on 71,836 father–son pairs (Fig. 1).

## 3 Statistical analysis

Son's height was adjusted for secular trends and the effect of age at examination by taking residuals from a regression of son's height on cubic splines of age at examination and date of birth (7 knots at percentiles of 2.5, 18.3, 34.2, 50, 65.8, 81.7 and 97.5; (Harrell, 2001); see Fig. A1 in Appendix). A similar adjustment was applied to father's height, where available. Within each quintile of son's adjusted height, we summarised fathers’ health-related characteristics at conscription examination, parents’ education levels and ages at their son's birth, and sons’ smoking (in a subset). Linear or logistic regression, as appropriate and without further adjustment, was used to examine the association of each of these variables with son's height.

### 3.1 Son's height as a proxy for parental height in the whole data set

Cox proportional hazards models were used to estimate hazard ratios (HR) for all-cause and cause-specific paternal and maternal mortality per standard deviation (SD, 6.49 cm) of son's height. Parental age was used as the time axis, so all models were adjusted for this. Additional adjustment was also made for the occupational SEI and educational level of the parent in question, which we will refer to collectively as socioeconomic position (SEP). Observations were right-censored at the earliest of the parent's date of death or emigration, or on 31st December 2004 (the end of follow-up). They were left-censored at the latest of the selected son's date of birth or 1st January 1961 (the start of follow-up). Hazard ratios for maternal and paternal mortality were compared by bootstrap resampling of those sons with valid maternal and paternal mortality (*N* = 973,440; 94% of those used for either parent). Linearity of associations was tested using fractional polynomials (Royston and Altman, 1994) of son's height in adjusted models. We also examined linearity of associations visually by plotting HR by tenths of son's height.

### 3.2 Analysis of the subsample of data with the father's own height

Using the subsample of data for which the father's own height at conscription examination was available (*N* = 71,836), the Cox proportional hazard models described above for fathers’ all-cause and cause-specific mortality were repeated. Using bootstrap resampling, HR from these models were compared with those from models using the same subsample, but using a father's own height in place of his son's height. To make the outcomes of all analyses comparable, HR were calculated per 6.49 cm (the SD of son's height) for both own height and son's height.

### 3.3 Two-sample instrumental variables analysis

Two-sample IV analysis (Angrist and Krueger, 1992; Didelez et al., 2010) allowed the estimation of HR for a father's all-cause and cause-specific mortality per 6.49 cm of his own height. This approach makes maximum use of the data available on fathers’ mortality (even when the father's height is not available, but his son's is), and by using son's height as an instrument, estimates the effect of own height on mortality in the absence of some sources of confounding. First father's height was regressed on son's height, adjusting for SEP, in the subset of data for which both father's and son's height were available. We divided the natural logarithm of the HR for mortality per SD of son's height (obtained by adjusted Cox models in the entire data set, as described above) by the adjusted regression coefficient for father's height against son's height. This ratio was exponentiated to give the two-sample IV hazard ratio. Confidence intervals were calculated using Taylor series expansions (Thomas et al., 2007). All statistical analyses were performed using Stata 11.2.

## 4 Results

There were 153,083 maternal deaths and 282,482 paternal deaths in the follow-up period. Table 1 shows characteristics of sons and their parents by quintiles of son's height. Son's height was strongly positively associated with father's height; the linear regression in Table 1 indicates that a father's unadjusted height at conscription examination increased by 3.04 cm (95% CI: 3.00, 3.08) for every one SD (6.49 cm) increment in his son's height (adjusted). Taller sons were less likely to be smokers. Their fathers were slightly older, with lower BMI and higher systolic blood pressure, were less likely to smoke and more likely to have completed more than 10 years of education. Mothers of taller sons were also older and had completed more education.

### 4.1 Association of son's height with paternal and maternal mortality

Tables 2 and 3 show the HRs for all-cause and cause-specific parental mortality per one SD increase in son's height. Son's height was inversely associated with all-cause mortality in both mothers (adjusted HR 0.97, 95% CI: 0.96, 0.97) and fathers (adjusted HR 0.98, 95% CI: 0.98, 0.98). Parents of taller sons had lower mortality from CVD, coronary heart disease (CHD), stroke, diabetes, respiratory disease, external causes and suicide. The negative associations of son's height with all-cause, CVD, CHD and respiratory disease mortality were considerably stronger for mothers than for fathers among sons with valid data on both parents.

Parental death from cancer was positively associated with son's height in mothers (adjusted HR 1.02, 95% CI: 1.02, 1.03) and fathers (adjusted HR 1.02, 95% CI: 1.01, 1.03). This weak positive association was the result of a mixture of positive and negative associations for specific cancers. Smaller numbers of deaths for specific cancers led to wide confidence intervals, but there was evidence for a negative association of son's height with maternal lung cancer, a positive association with paternal prostate cancer, and a positive association with breast, colon and kidney cancers in both parents. With the exception of lung cancer (negative association for mothers and null for fathers), HRs for cancer mortality did not differ between mothers and fathers. Son's height was also positively associated with death from aortic aneurysm in fathers, but not in mothers.

**Fig. 2:**
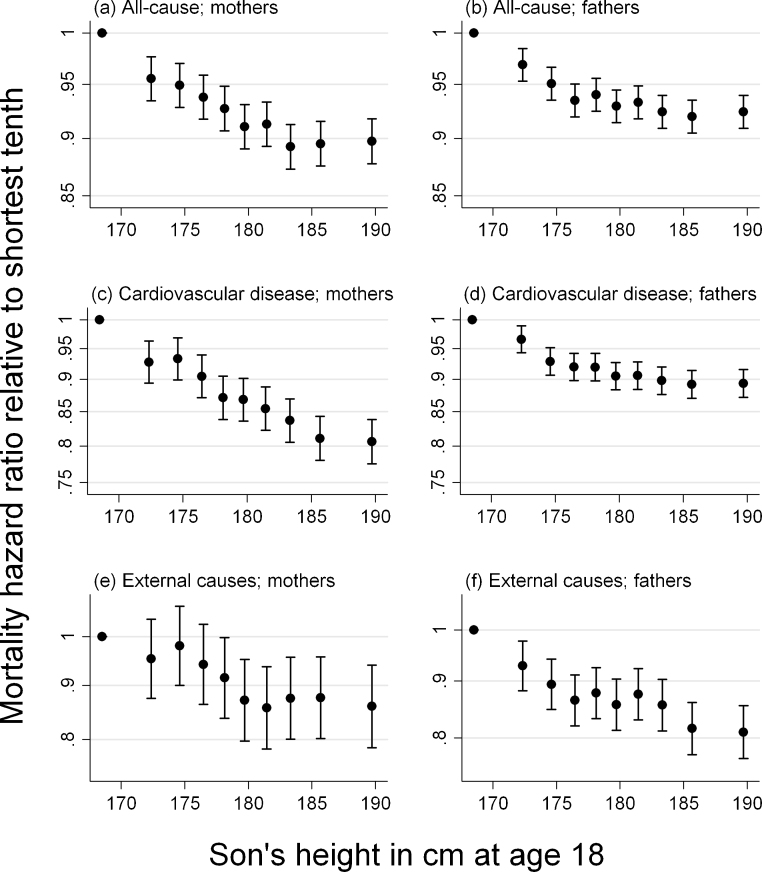
Hazard ratios (with 95% confidence intervals) for mortality from (a and b) any cause, (c and d) cardiovascular disease and (e and f) external causes, adjusted for age and socio-economic position, among the parents of Swedish men born between 1951 and 1980 and subjected to conscription examination. The hazard ratio for each tenth of son's height (adjusted for secular trends and age at examination) is plotted at its median value, with the first tenth as the reference group. Plots for all mortality causes, and by father's own height, are available in Fig. A2 of Appendix.

Associations generally attenuated slightly towards the null with additional adjustment for SEP. The positive associations with cancer (both parents) and aortic aneurysm (fathers) provided exceptions to this, being generally a little stronger after adjustment. Fractional polynomial models found substantial evidence (*P* < 0.001) in both parents for nonlinearity in the relationships of all-cause and CVD mortality with son's height, and weaker support (0.05 < *P* < 0.10) for CHD in mothers, and external causes and suicide among fathers. Plots of mortality rates against tenths of son's height (see Fig. A2 in Appendix) suggest that the statistical significance owes more to the considerable statistical power available than to the magnitude of the nonlinearity, and that the linear assumption is not unreasonable in these analyses. The slight nonlinearity of all-cause, CVD and CHD mortality in relation to son's height appeared to be due to the very tallest sons, whose parents experienced higher mortality rates than would be expected from the overall negative trend (Fig. 2a–d). By contrast, the non-linearity in paternal suicide and death from all external causes appeared to be driven by particularly high mortality rates among the fathers of the very shortest men.

### 4.2 Son's height as an instrument for father's height

The regression coefficient used in the two-sample IV analyses (with adjustment for SEP, and both heights adjusted for their respective date of birth and age at examination and scaled by a SD of 6.49 cm) was only slightly lower than the unadjusted figure (Table 1), at 0.46 (95% CI: 0.45, 0.47); equivalent to 2.99 cm per SD (95% CI: 2.95, 3.03). Two-sample IV estimates for associations of a father's own height with mortality (Table 3) were further from the null than were the corresponding Cox estimates, with wider confidence intervals. Nonetheless, they led to similar conclusions for each cause of death.

Table 4 shows the association of all-cause and cause-specific paternal mortality with son's height and the father's own height within the subset of father–son pairs for which the father's height was available (Fig. 1), for those causes with at least 50 deaths in the subset. The associations of son's height with fathers’ outcomes in this subgroup were generally similar to those reported in Table 3 for the whole analysis cohort. For all outcomes except lung cancer, the associations were stronger when the father's own height, rather than his son's, was used although 95% confidence intervals for the difference between the two HR (from 100 bootstrap resamples) invariably overlapped zero.

## 5 Discussion

In this study we used a son's height as an IV to obtain unbiased estimates of the associations between height and mortality. The parents of taller sons had lower mortality overall, and from CVD, CHD, stroke, diabetes, respiratory disease, external causes and suicide. Son's height was positively associated in both parents with mortality from cancer, and among fathers only, with mortality from aortic aneurysm.

### 5.1 Strengths and limitations of the study

Our study benefits from a very large, prospective sample, comprising the parents of an almost complete population cohort of young Swedish men. The only major section of the population excluded from our study is those who do not have a son, or whose son does not survive to conscription age. The sample size gives us the statistical power to investigate rarer causes of death, with greater precision, than most previous studies. For example, the sample size here is about twice that of a recent collaborative study combining mortality and own height data from 113 prospective studies (Wormser et al., in press). The use of a son's height rather than the parent's own height also contributed to the large sample size, since the follow-up time required to record large numbers of deaths is reduced by the length of a generation.

Fathers who had had their own height recorded at conscription examination were a young, recent subsample, representing only 7% of the larger sample whose son's height was measured. Causes of death affecting mainly younger men, particularly external causes, dominate this subsample, making it difficult to compare results with those in the entire sample.

By using a son's height as an instrument for parents’ heights, we believe we have avoided confounding by existing parental ill health as comprehensively as possible. IVs rely on assumptions, and as is the case for all methods of causal inference using observational data, some of these are untestable (Didelez et al., 2010). An instrument should be strongly associated with the variable it replaces. Most studies have found maternal and paternal height to be strongly and similarly associated with offspring height (Luo et al., 1998; Pearson and Lee, 1903; Solomon et al., 1983), and we were able to confirm this for paternal height in the present study population (we did not have data on maternal height). An instrument should also be independent of the unmeasured confounding. A son's height might be influenced by behavioural, genetic or socioeconomic factors confounding mortality and height in the parent, although such associations may be weaker for the son's height than for the parent's own height. Finally, there must be no pathway associating the outcome and the instrument, except for those passing through the exposure. Apart from the potential association between the instrument and the confounders discussed above, it is difficult to conceive of other such pathways.

### 5.2 How do we interpret the results?

Son's height is likely to be less influenced by the potential confounding effect of adult ill health on both height (usually shrinkage) and mortality. The similarity between the results we obtained using (i) a father's own height, (ii) a son's height, and (iii) a son's height as an instrument for his father's height, suggest that confounding by existing ill-health does not greatly influence the associations between own height and mortality reported here and elsewhere. The use of son's height as an IV only partially corrects for confounding by other factors, such as socioeconomic position or genetic pleiotropy, because these are strongly associated between parents and offspring. Such confounders remain possible drivers of our observed associations between a son's height and parental mortality.

Our observation that son's height is more strongly predictive of maternal than paternal mortality may be due to (i) stronger association between a son's height and his mother's height, than his father's height (e.g. due to a greater degree of shared environment, or mis-assigned paternity, although most studies (Luo et al., 1998; Pearson and Lee, 1903; Solomon et al., 1983) find that such associations do not differ greatly between parents), (ii) stronger unmeasured confounding of maternal than paternal mortality with son's height (e.g. through an intra-uterine effect of maternal smoking) or (iii) a stronger association between mortality and own height among women than among men. The stronger prediction of mothers’ mortality did not apply for the few outcomes we tested which were positively associated with son's height. This would support the second of the three interpretations above, although a more robust test of these alternatives would require data on each mother's own height. A recent meta-analysis of 113 prospective studies (Wormser et al., in press) found that the association of height with CHD in women was somewhat stronger than the same association in men, but noted no strong evidence of gender differences for other outcomes.

Adjusting for SEP tended to increase the strength of positive associations (i.e. aortic aneurysm, most cancers) and attenuate the negative associations. This is probably because parents of higher SEP have both lower mortality rates and taller children due to healthier lifestyles. Data on other potentially important confounders were very limited. It is likely, for example, that the negative association between son's height and maternal mortality from lung cancer (Table 2) is largely due to confounding by smoking, with lower levels of smoking among the mothers of taller sons. However, we do not have information on smoking among mothers to test this.

The relationships we observed between cause-specific mortality rates and son's height were close to linear on the logarithmic scale assumed by Cox regression (see Fig. A2 in Appendix). Deviations from this linear trend suggested a detrimental effect on survival of being extremely tall or short, beyond the trend seen in the population as a whole. Some studies (Engeland et al., 2003; Koch, 2011) have suggested that the association between height and all-cause mortality may be positive among the very tallest women, but not men. Within the tallest tenth of sons, we found that all-cause mortality in both parents was unrelated, or perhaps slightly positively associated, with son's height, although there was no indication of this among the limited data available for fathers’ own height (see Fig. A3 in Appendix).

### 5.3 Comparison with other studies

To the best of our knowledge, this is the first study to use son's height as an instrument for parents’ height. Previous studies, including the very large emerging risk factor collaboration of over a million individuals (Wormser et al., in press) have found inverse associations between a subject's own height and their mortality from all causes (Engeland et al., 2003; Jousilahti et al., 2000; Song and Sung, 2008), CVD (Cook et al., 1994; Lee et al., 2009; Paajanen et al., 2010) and external causes (Koch, 2011; Lee et al., 2009; Song and Sung, 2008), and positive associations with mortality from most cancers (Batty et al., 2010; Green et al., 2011; Gunnell et al., 2001). Our findings, using son's height as an instrumental variable, are consistent with these results.

## 6 Conclusions

We found that height at age 18 was inversely associated with mortality from all-causes, CVD, CHD, stroke, diabetes, respiratory disease, external causes and suicide. It was positively associated with mortality from cancer and, in fathers, from aortic aneurysm. Using son's height as an instrumental variable for parent's height allowed us to explore whether conventional approaches to examining the association of height with mortality are biased by ill-health resulting in shrinkage. The associations we found between paternal mortality and height were similar whether an instrumental variables approach or a conventional multivariable approach was used. This suggests that shrinkage due to ill-health was not an important source of confounding, and increases the confidence with which we can interpret conventional analyses of mortality–height associations, at least when height has been measured in young adulthood. Our approach may also have reduced the effect of other unmeasured confounding, but the extent of this is difficult to judge. Further studies are required to examine the extent to which other sources of confounding may explain these results and the likelihood of other (biological) mechanisms for these associations.

## Figures and Tables

**Table 1:** Characteristics of sons and parents according to quintiles of son's height, adjusted for secular trends and age at examination. Linear and logistic regressions of characteristics against son height are conducted per standard deviation (6.49 cm) of adjusted son's height.

Subject	Measurement	Quintile of son's height					Regression coefficient or OR (95% CI)	*N*
		1st	2nd	3rd	4th	5th		
Sons	Mean unadjusted height^a^ (cm)	170.3	175.8	179.2	182.7	188.4		1,036,963
	Proportion smokers^a^ (%)	64.0	60.1	59.3	58.4	54.1	0.87^b^ (0.85, 0.90)	29,541

Fathers	Mean unadjusted height^a^ (cm)	174.2	176.8	178.4	180.1	182.7	3.04 (3.00, 3.08)	73,010
	Proportion smokers^a^ (%)	66.2	64.2	64.2	63.5	59.7	0.91^b^ (0.89, 0.94)	16,967
	Mean age at son's birth (years)	30.3	30.4	30.5	30.6	30.8	0.16 (0.15, 0.17)	997,110
	Mean BMI^a^ (kg m^−2^)	21.3	21.3	21.3	21.2	21.2	−0.04 (−0.06, −0.02)	72,991
	Mean systolic BP^a^ (mm Hg)	126.4	126.6	126.8	126.7	127.3	0.32 (0.24, 0.40)	72,997
	Proportion educated >10 years (%)	49.9	52.2	53.6	55.4	57.8	1.12^b^ (1.12, 1.13)	949,032

Mothers	Mean age at son's birth (years)	27.0	27.2	27.4	27.5	27.8	0.26 (0.25, 0.27)	1,013,293
	Proportion educated >10 years (%)	49.9	52.4	53.9	55.2	58.1	1.12^b^ (1.12, 1.13)	994,098

*Abbreviations*: BMI: body mass index; BP: blood pressure; OR: odds ratio; CI: confidence interval.

a Measured at time of conscription examination.

b Logistic regression; odds ratios (exponentiated coefficients) are presented.

**Table 2:** Cox models for maternal mortality per standard deviation (6.49 cm) of son's height, adjusted for secular trends and age at examination.

Cause of death	Deaths	Age adjusted		Age and SEP adjusted		Comparison between parents^a^
		Hazard ratio	95% CI	Hazard ratio	95% CI	
All cause	153,083	0.95	0.94, 0.95	0.97	0.96, 0.97	0.015
Cardiovascular disease	50,922	0.91	0.91, 0.92	0.94	0.93, 0.95	<0.001
Coronary heart disease	24,020	0.87	0.86, 0.88	0.90	0.88, 0.91	<0.001
Aortic aneurysm	1529	1.01	0.96, 1.06	1.02	0.97, 1.07	0.085
Stroke	14,194	0.94	0.92, 0.95	0.96	0.94, 0.97	0.283
Diabetes	2603	0.82	0.79, 0.86	0.85	0.82, 0.89	0.037
Respiratory diseases	7845	0.84	0.82, 0.86	0.86	0.84, 0.88	<0.001
Cancer	62,307	1.01	1.00, 1.02	1.02	1.02, 1.03	0.907
Lung cancer	7668	0.92	0.89, 0.94	0.92	0.90, 0.94	<0.001
Breast cancer	11,435	1.07	1.05, 1.09	1.08	1.06, 1.10	0.225
Colon cancer	4751	1.03	1.00, 1.06	1.05	1.02, 1.08	0.291
Stomach cancer	2456	0.97	0.93, 1.01	1.00	0.96, 1.04	0.929
Kidney cancer	1983	1.01	0.96, 1.05	1.03	0.98, 1.07	0.492
External causes	9453	0.93	0.91, 0.95	0.95	0.93, 0.97	0.647
Suicide	3821	0.95	0.92, 0.98	0.97	0.94, 1.00	0.378

*Abbreviations*: SEP: socioeconomic position (includes education and occupational socioeconomic index); CI: confidence interval. *N* = 1,013,293 mothers at risk of mortality.

a *P*-value for the comparison of age and SEP-adjusted hazard ratios between fathers (Table 3) and mothers. Calculated from 100 bootstrap resamples of sons with valid data on both parents.

**Table 3:** (i) Cox models for paternal mortality per standard deviation (6.49 cm) of son's height and (ii) adjusted two-sample IV models for paternal mortality against own height, using son's height as an instrument.

Cause of death	Deaths	Son's height, age adjusted		Son's height, age and SEP adjusted		Two-sample IV, age and SEP adjusted	
		Hazard ratio	95% CI	Hazard ratio	95% CI	Hazard ratio	95% CI
All cause	282,482	0.96	0.96, 0.97	0.98	0.98, 0.98	0.96	0.95, 0.96
Cardiovascular disease	127,365	0.95	0.95, 0.96	0.97	0.96, 0.97	0.93	0.92, 0.95
Coronary heart disease	81,776	0.94	0.93, 0.95	0.96	0.95, 0.97	0.91	0.90, 0.93
Aortic aneurysm	5003	1.07	1.04, 1.10	1.07	1.04, 1.10	1.17	1.10, 1.24
Stroke	21,511	0.93	0.92, 0.94	0.95	0.93, 0.96	0.89	0.86, 0.91
Diabetes	4280	0.90	0.87, 0.93	0.92	0.89, 0.94	0.83	0.77, 0.88
Respiratory diseases	14,021	0.93	0.91, 0.94	0.95	0.93, 0.96	0.88	0.85, 0.92
Cancer	79,521	1.01	1.01, 1.02	1.02	1.01, 1.03	1.05	1.03, 1.06
Lung cancer	15,053	0.97	0.96, 0.99	0.99	0.97, 1.01	0.98	0.94, 1.01
Breast cancer	70	1.29	1.02, 1.63	1.29	1.02, 1.63	1.74	1.04, 2.89
Prostate cancer	12,714	1.02	1.00, 1.03	1.02	1.00, 1.03	1.04	1.00, 1.08
Colon cancer	5779	1.02	0.99, 1.04	1.02	0.99, 1.04	1.04	0.98, 1.10
Stomach cancer	4930	0.98	0.95, 1.00	1.00	0.97, 1.03	1.00	0.94, 1.06
Kidney cancer	3513	1.05	1.01, 1.08	1.06	1.02, 1.09	1.13	1.05, 1.21
External causes	25,662	0.92	0.91, 0.93	0.95	0.94, 0.96	0.89	0.87, 0.91
Suicide	9569	0.93	0.91, 0.95	0.96	0.94, 0.98	0.91	0.87, 0.95

*Abbreviations*: SEP: socioeconomic position (includes education and occupational socioeconomic index); IV, instrumental variables; CI: confidence interval. *N* = 997,110 fathers at risk of mortality and 71,836 father–son pairs with recorded height for the two-sample IV model. All heights were adjusted for secular trends and age at examination.

**Table 4:** Fathers’ mortality per standard deviation (6.49 cm) of height at conscription (son's or own), adjusted for secular trends and age at examination.

Cause of death	Deaths	Son's height^a^, age adjusted		Son's height^a^, age and SEP adjusted		Own height^a^, age adjusted		Own height^a^, age and SEP adjusted	
		Hazard ratio	95% CI	Hazard ratio	95% CI	Hazard ratio	95% CI	Hazard ratio	95% CI
All cause	2553	0.92	0.89, 0.96	0.95	0.92, 0.99	0.89	0.85, 0.92	0.93	0.89, 0.96
Cardiovascular disease	450	0.90	0.82, 0.98	0.92	0.84, 1.01	0.86	0.78, 0.95	0.90	0.82, 1.00
Coronary heart disease	248	0.88	0.78, 1.00	0.90	0.80, 1.03	0.77	0.68, 0.88	0.80	0.70, 0.92
Stroke	94	0.85	0.69, 1.04	0.87	0.71, 1.07	0.83	0.67, 1.03	0.87	0.70, 1.08
Cancer	485	1.04	0.95, 1.14	1.06	0.97, 1.16	1.06	0.97, 1.17	1.08	0.98, 1.19
Lung cancer	69	1.15	0.91, 1.46	1.17	0.93, 1.49	1.08	0.85, 1.38	1.12	0.87, 1.43
External causes	1147	0.89	0.84, 0.95	0.93	0.88, 0.99	0.86	0.81, 0.91	0.90	0.85, 0.96
Suicide	512	0.86	0.79, 0.94	0.90	0.82, 0.98	0.82	0.75, 0.90	0.86	0.79, 0.94

*Abbreviations*: SEP: socioeconomic position (includes education and occupational socioeconomic index); CI: confidence interval.

a Separate analyses were made using either each father's own height, or a son's height. Data were restricted to father–son pairs where both heights were available (*N* = 71,836) and to death causes with at least 50 deaths.
