# Supplementary material for: Associations of mortality with own height using son's height as an instrumental variable
Source: Econ Hum Biol. 2013 Jul;11(3):351–9. doi: 10.1016/j.ehb.2012.04.003 (PMC3685807; doi:10.1016/j.ehb.2012.04.003)

**Appendix A. Supplementary data for "Associations of mortality rates with height using son’s height as an instrumental variable" by David Carslake, Abigail Fraser, George Davey Smith, Margaret May, Tom Palmer, Jonathan Sterne, Karri Silventoinen, Per Tynelius, Debbie A Lawlor and Finn Rasmussen.**

Table A1. International classification of diseases (ICD) codes included for each cause of death. An asterisk indicates any code beginning with the indicated letter.

| Cause of death |  | ICD 7 (1961-1968) |  | ICD 8 (1969-1986) |  | ICD 9 (1987-1996) |  | ICD 10 (1997-present) |
| --- | --- | --- | --- | --- | --- | --- | --- | --- |
| All cause |  | all |  | all |  | all |  | all |
| Circulatory system disease |  | 3300-3399; 4000-4549; 4560-4689; 7820-7829 |  | 3900-4441; 4444-4589; 7820-7829 |  | 3900-4599 |  | I* |
| Coronary heart disease |  | 4200-4202; 4220-4222; 4500-4509 |  | 4100-4149; 4922 |  | 4100-4149; 4922 |  | I200-I259; I516 |
| Aortic aneurysm |  | 4510-4519 |  | 4410-4419 |  | 4410-4419 |  | I710-I719 |
| Stroke |  | 3300-3349; 3520-3529 |  | 2930-2931; 4300-4389; 3440-3449 |  | 2904; 3420-3429; 3440-3449; 4300-4389 |  | I600-I699; G450-G459 |
| Diabetes |  | 2600-2609 |  | 2500-2509 |  | 2500-2509 |  | E100-E149 |
| Respiratory diseases |  | 2400-2409; 2410-2419; 4700-5279 |  | 4600-5199 |  | 4600-5199 |  | J* |
| Cancer |  | 1400-2079; 2923; 2941-2949 |  | 1400-2099 |  | 1400-2089; 2384; 2898 |  | C* |
| Lung cancer |  | 1620-1639 |  | 1620-1639 |  | 1620-1639 |  | C330-C349 |
| Breast cancer |  | 1700-1709 |  | 1740-1749 |  | 1740-1759 |  | C500-C509 |
| Prostate cancer |  | 1770-1779 |  | 1850-1859 |  | 1850-1859 |  | C610-C619 |
| Colon cancer |  | 1530-1539 |  | 1530-1539 |  | 1530-1539 |  | C180-C189 |
| Stomach cancer |  | 1510-1519 |  | 1510-1519 |  | 1510-1519 |  | C160-C169 |
| Kidney cancer |  | 1800-1801 |  | 1890-1892 |  | 1890-1899 |  | C640-C669 |
| External causes |  | 8000-9999 |  | 8000-9999 |  | 8000-9999 |  | V*; W*; X*; Y* |
| Suicide |  | 9700-9799 |  | 9500-9590 |  | 9500-9599 |  | X600-X849 |

Figure A1. Details from the adjustment of height for age at examination and date of birth. Age at examination was centred around 18 years and date of birth was centred around 1955 for measured fathers and 1965 for sons. Height values were then adjusted by taking residuals from a linear regression of height against cubic splines (7 knots at percentiles of 2.5, 18.3, 34.2, 50, 65.8, 81.7 and 97.5) of date of birth and age at examination. Note the different scale on the y axis of (c). We interpret the decline in height in the few men measured beyond age 20 as being due to men with health problems delaying conscription, rather than individuals shrinking.


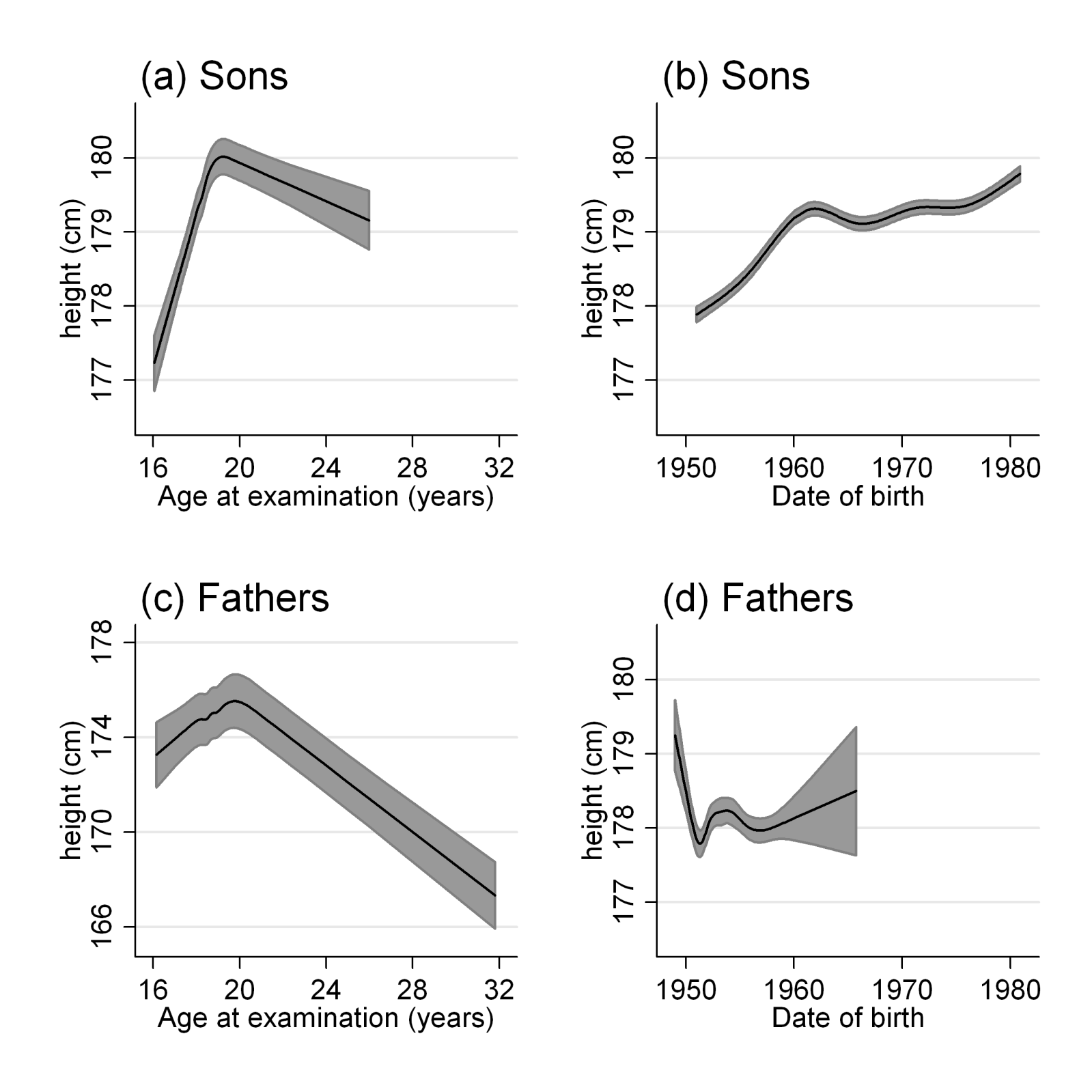


Figure A2. Hazard ratios (with 95% confidence intervals) for all-cause and cause-specific parental mortality, adjusted for parental age and socio-economic position. The hazard ratio for each tenth of (a-c) son's or (d) father's own height (adjusted for secular trends and age at examination) is plotted at its median value, with the first tenth as the reference class. There were (a) 1,013,293 mother-son pairs and (b) 997,110 father-son pairs in the full data set, and (c, d) 71,836 father-son pairs in the subset of data for which the father's height was known.

d) Fathers' mortality vs own height (subset)

c) Fathers' mortality vs a son's height (subset)

b) Fathers' mortality vs a son's height (all data)

a) Mothers' mortality vs a son's height (all data)

c) Fathers' mortality vs a son's height (subset)

d) Fathers' mortality vs own height (subset)

b) Fathers' mortality vs a son's height (all data)

a) Mothers' mortality vs a son's height (all data)

d) Fathers' mortality vs own height (subset)

c) Fathers' mortality vs a son's height (subset)

b) Fathers' mortality vs a son's height (all data)

a) Mothers' mortality vs a son's height (all data)

Figure A3. Hazard ratios (with 95% confidence intervals) for all-cause mortality per standard deviation (6.49 cm) of son's (a,b) or own (c) height, for tenths of the height distribution, plotted at the median height for each tenth. The horizontal dotted line and shaded area represent the overall hazard ratio and 95% confidence intervals.


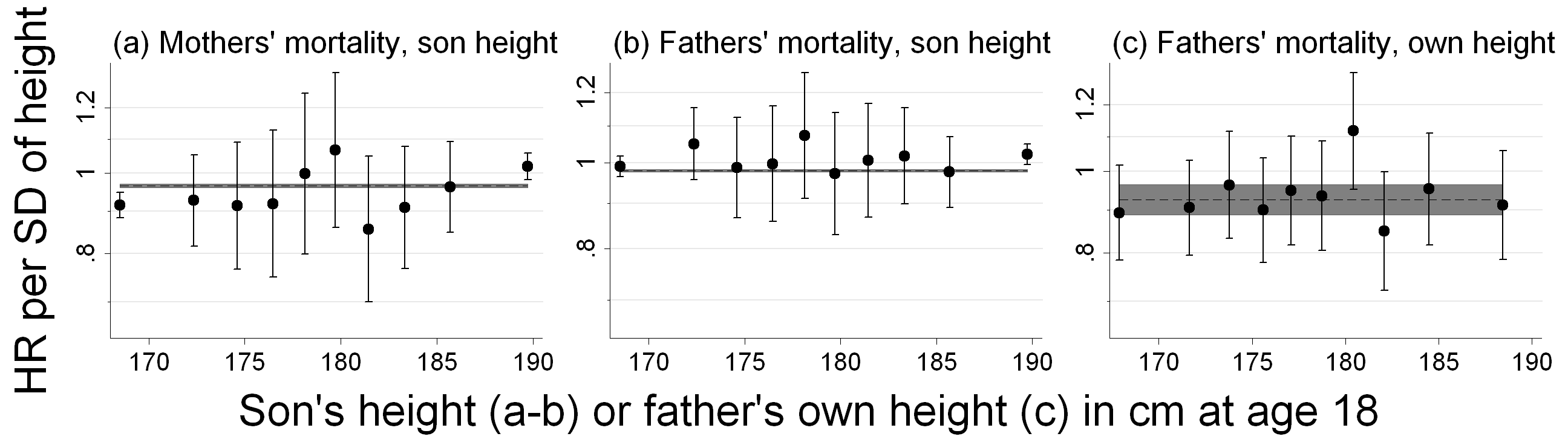

Supplement: Supplementary file 1 [file mmc1.docx]
